# Performance evaluation of large language models in the diagnosis of emergency internal medicine diseases: a retrospective study

**DOI:** 10.3389/fpubh.2026.1780425

**Published:** 2026-05-08

**Authors:** Jintao Wei, Shouyin Jiang, Ting Yin, Jiangchun Ma, Qiang Li, Yu Tian, Mengting Yan, Zhuyi Shen, Xiangkang Lv, Ximei Ma, Shanxiang Xu, Mao Zhang

**Affiliations:** 1 Department of Emergency Medicine, Second Affiliated Hospital, Zhejiang University School of Medicine, Hangzhou, China; 2 Key Laboratory of the Diagnosis and Treatment of Severe Trauma and Burn of Zhejiang Province, Hangzhou, China; 3 Zhejiang Province Clinical Research Center for Emergency and Critical Care Medicine, Hangzhou, China; 4 National Emergency Medical Rescue Base, Hangzhou, China; 5 Department of Cardiology, The Second Affiliated Hospital, School of Medicine, Zhejiang University, Hangzhou, China; 6 Department of Neurosurgery, Zhejiang Hospital, Hangzhou, China; 7 Engineering Research Center of EMR and Intelligent Expert System, Ministry of Education, Key Laboratory for Biomedical Engineering of Ministry of Education, College of Biomedical Engineering and Instrument Science, Zhejiang University, Hangzhou, China; 8 Nursing Department, The Second Affiliated Hospital of Zhejiang University School of Medicine, Hangzhou, China; 9 Hangzhou Institute for Advanced Study, University of Chinese Academy of Sciences, Hangzhou, China

**Keywords:** DeepSeek-V3, diagnostic accuracy, emergency department junior physicians, GPT-4o, large language model

## Abstract

**Objective:**

Medical domain large language models (LLMs) exhibit verified clinical decision-support capabilities in simulated case analyses and standardized tests, yet their diagnostic efficacy in real-world emergency settings remain insufficiently explored. This study evaluates the diagnostic performance of 5 mainstream LLMs (ChatGPT-4o, Gemini-2.0, Grok3, DeepSeek-V3, Doubao) against emergency department junior physicians (EDJP) on real-world emergency internal medicine cases.

**Methods:**

A single-center retrospective analysis design was conducted. 154 anonymized emergency internal medicine patients of the Second Affiliated Hospital of Zhejiang University School of Medicine from January to May 2025 were included, covering common acute diseases of multiple systems. 15 EDJPs and 5 LLMs were selected to diagnose the cases, respectively. The main diagnostic accuracy, comprehensiveness of differential diagnosis, and response time were used as evaluation indicators. Non-parametric tests were used for statistical analysis.

**Results:**

(1) Main diagnostic accuracy: DeepSeek-V3 (90.0%), ChatGPT-4o (86.0%), and Grok3 (86.0%) were significantly higher than that of EDJP (77.5%, *p* < 0.05); in the subgroup of respiratory system diseases, Gemini-2.0 and DeepSeek-V3 performed better (*p* < 0.05). (2) Comprehensiveness of differential diagnosis: The scores of all LLMs were significantly higher than that of EDJP (*p* < 0.05), and the medians of DeepSeek-V3, Gemini-2.0, and Grok3 reached 5.0 points. (3) Response time: LLMs (6.3–14.0 s) were significantly faster than EDJP (360.2 s, *p* < 0.05), and Doubao had the fastest response. The inter-rater reliability was good (ICC: 0.617–0.899).

**Conclusion:**

This retrospective study shows that LLMs outperformed EDJPs in diagnostic accuracy, differential diagnosis comprehensiveness and response efficiency for emergency internal medicine diseases, demonstrating significant potential for clinical decision support. Subsequent efforts will focus on exploring how to effectively integrate into physician-led collaborative workflows to enhance emergency care quality and efficiency.

## 1 Introduction

In recent years, large language models (LLMs) and artificial intelligence (AI) systems have exhibited remarkable potential in the medical domain, yielding promising preliminary results in health economics, public health, medical education, biomedicine, and clinical decision support (1–4). Notably, untrained LLMs have demonstrated capabilities to pass all stages of the U.S. Medical Licensing Examination, generate high-quality patient communication content, and address complex cross-professional medical issues (5). However, their performance, safety, and practical integration in clinical settings require further systematic evaluation.

Emerging next-generation LLMs-such as those released by DeepSeek, OpenAI, and other institutions—represent a new paradigm in AI development (6, 7). For instance, models like GPT-4o, Gemini, Grok3, DeepSeek-V3, and Doubao, trained on massive biomedical and clinical corpora and equipped with billions of parameters, can already support tasks such as triage, chronic disease management, decision-making support, and patient education (8). By modeling extensive medical knowledge, these models can quickly generate detailed differential diagnoses, offsetting human clinicians’ limitations in information integration and cognitive load. For example, LLM-generated handover records outperform clinician-written ones in readability and completeness, offering workflow optimization insights for emergency departments (ED) (9).

Controversies persist regarding real-world diagnostic efficacy. While studies show surgical recommendations from ChatGPT-4o, Gemini 2.0, and DeepSeek-V3 align with expert advice. Masanneck et al. (10) found that indicates LLMs perform only as well as untrained clinicians in emergency triage, with over-classification tendencies—highlighting the need for domain-specific optimization in high-risk ED environments. A randomized controlled trial shows that the independent diagnostic accuracy of LLMs achieve higher independent diagnostic accuracy than clinicians yet integrating LLM results did not significantly improve overall clinician performance (11). A New England Journal of Medicine study (12) involving 100 cases reported low diagnostic accuracy rates of 35% (DeepSeek-R1) and 39% (GPT-4), underscoring integration challenges. In addition, Grok3, released by xAI in June 2024 as a publicly accessible platform, has competitive performance and multimodal capabilities, and emphasizes real-time reasoning ability and adaptability to open domains. These findings highlight challenges in effectively integrating high-performance LLMs with clinical practice. EDs, as frontlines for critically ill patients, face escalating pressures from high workload, complexity, and rapid turnover. Delayed diagnosis increases serious adverse event risks by 12% per hour (13), while 38% of ED patients present overlapping symptoms, and 42% of critically ill patients have initial complaints inconsistent with final diagnoses (14).

Early identification of severe sepsis/septic shock risks simplifies subsequent management, and ED diagnosis reliance on structured data (medical records, test results) aligns with LLMs’ reasoning strengths (15). However, over 90% of AI decision systems remain preclinical, with only 5% of studies using real patient data, lacking systematic evaluation of mainstream LLMs in real ED diagnosis (16).

Emergency department junior physicians (EDJP), pivotal in initial assessment and treatment, show greater AI tool acceptance but face diagnostic variability due to limited experience-particularly in time-pressured settings (15). Notably, EDJP often exhibit higher acceptance and reliance on intelligent tools than senior physicians, offering a unique perspective on LLMs’ real-world utility. Systematic evaluation of LLMs in EDJP’ daily diagnosis and treatment objectively assesses model applicability and reliability, provides practical AI-assisted solutions for primary medical institutions, and promotes standardized application and popularization of AI in emergency medicine (17).

This study adopts a retrospective approach to evaluate 5 mainstream reasoning LLMs (ChatGPT-4o, Gemini-2.0, Grok3, DeepSeek-V3, Doubao) using real ED internal medicine consultation cases, focusing on primary diagnosis accuracy, differential diagnosis comprehensiveness, and response efficiency. It aims to fill real-world evidence gaps and provide empirical support for clinical implementation. Based on real emergency internal medicine consultation cases, a comparative evaluation is carried out from three core dimensions: the accuracy of the primary diagnosis, the comprehensiveness of differential diagnosis, and the efficiency of response time. This study aims to fill the gap in real-world evaluation evidence, provide empirical support for the clinical implementation.

## 2 Materials and methods

### 2.1 Research design and participants

This study was designed as a single-center retrospective analysis aiming to compare the performance of 5 mainstream domestic and international LLMs-ChatGPT-4o (Open AI, San Francisco, CA, USA), Gemini-2.0 (Google DeepMind, London, UK), Grok3 (xAI, San Francisco, CA, USA), DeepSeek-V3 (DeepSeek Inc., Hangzhou, China), and Doubao (Byte Dance, Beijing, China)-with EDJP across main diagnosis accuracy, differential diagnosis comprehensiveness, and response time. All LLMs were accessed via their respective official web interfaces, and the experiments were conducted between September 1 and October 1, 2025.

The study initially included 164 adult patients who visited the Emergency Department of the Second Affiliated Hospital of Zhejiang University from January 2025 to May 2025 and were unplanned hospitalized due to internal medicine diseases (5 cases were excluded due to incomplete or missing medical records, non-internal medicine diseases and other reasons, and another 5 cases were reserved for testing). Finally, a total of 154 cases were included in the study. The cases covered common acute internal medicine diseases of the nervous system, circulatory system, respiratory system, digestive system, urinary system, and other systems. 15 EDJPs who had completed the emergency department resident training and worked for 3–5 years were included. Each person was randomly and evenly assigned 10 cases for diagnosis. All 15 EDJPs were fully blinded to the final diagnosis, with no access to discharge outcomes or prior case exposure; diagnostic assessments for EDJPs and LLMs were completed synchronously and independently.

### 2.2 Inclusion and exclusion criteria

Inclusion Criteria: Age ≥ 18 years old; Unplanned emergency department visit on the same day due to internal medicine diseases based on the International Classification of Diseases, 10th Revision (ICD-10); The current emergency department visit is not a planned hospitalization.

Exclusion Criteria: Patients transferred from the outpatient department to the emergency department; patients with non-internal medicine conditions (including surgical diseases, trauma); internal medicine patients who received hospitalization certificates, examination forms, or medication prescriptions; patients with incomplete medical records or missing key information.

This is because retrospective inaccessibility of physical examination photos-particularly for trauma patients—resulted in incomplete data that could compromise study validity.

### 2.3 Data processing

The collected content includes demographic information (age, gender), medical history, medication history, laboratory tests, test results, and complete diagnostic records. All data are analyzed after anonymization, strictly following the Declaration of Helsinki and the principles of data privacy protection. The research protocol has been approved by the Ethics Committee of the Second Affiliated Hospital of Zhejiang University the complete medical records of patients are extracted through the hospital’s electronic medical record system, and the original medical records are structurally transcribed to compensate for the insufficient ability of some LLMs to process images and unstructured information. Microsoft Word is used to organize the structured data, and two researchers independently cross-check to ensure the accuracy and consistency of the input information.

### 2.4 Testing and application of LLMs

To ensure the consistency of experimental operations, all case-related queries were input into the LLMs by a single researcher (MTY). Each case was processed in an independent chat session to prevent the models from applying any incremental learning to subsequent cases. Notably, the retrieval-augmented generation technique was not incorporated into the LLMs-based analysis pipeline. The medical records were, respectively, input into the 5 LLMs and the EDJP group. All LLMs were accessed via the web, and the experiments were conducted between September 1 and October 1, 2025.

All case data (history, physical examination, tests, and imaging) were iteratively transformed into a uniform prompt: “I am an emergency department physician managing an emergency patient. Based on the provided clinical data (history, physical examination, tests, imaging). Please make a diagnosis according to the ICD-10 disease classification, make the most likely diagnosis, and divide it into the main diagnosis and secondary diagnosis (no explanation required).” And record the time taken by EDJP and LLMs to complete the diagnostic record. Two examples of representative cases with corresponding input information provided to the LLMs are presented (Supplementary Table S1).

### 2.5 Diagnostic criteria and evaluation system

The diagnostic gold standard was defined as the final clinical diagnosis documented in the front sheet of the medical record. Diagnoses were classified and grouped according to ICD-10 coding. Primary diagnosis: refers to the primary disease or clinical manifestation that led the patient to seek emergency treatment; Secondary diagnosis: refers to other co-existing diseases or clinical conditions related to this visit.

The accuracy score of the primary diagnosis is divided into 0–2 points. 2 points indicated a correct diagnosis, 1 point indicated a partially correct diagnosis, and 0 points denoted an incorrect diagnosis. Specifically, 2 points were awarded for a correct diagnosis that included all major diagnoses identified at admission, regardless of minor diagnoses. A score of 1 point was given for a partially correct diagnosis, which can occur in 2 scenarios: either the major diagnosis is nearly correct, and the subtle differences would not have impacted treatment, or there is suspicion of multiple major differential diagnoses, with one being correct and the others incorrect. However, 0 points were assigned when all major diagnoses were incorrect. Minor diagnoses were excluded from scoring, as they did not exert a significant impact on the patient’s clinical condition.

The comprehensiveness of differential diagnosis is evaluated using the Likert 5-point scale, with the scoring criteria defined within the top 5 diagnostic considerations as follows: 5 points. The differential diagnosis includes all reasonable diagnoses; 4 points. The differential diagnosis includes most diagnoses, but some are missing; 3 points. The differential diagnosis includes some candidates, but a few are missing; 2 points. The differential diagnosis includes some of the main diagnoses, but many are missing; 1 point. The differential diagnosis misses all the main diagnoses (18). We have provided the complete diagnoses and scores for 2 medical cases, which are presented (Supplementary Table S2).

To evaluate the consistency and reliability of LLMs, we randomly selected 5 cases from the included cases and presented them to the artificial intelligence models 3 times in independent conversations. If the model provides basically the same main diagnosis and a similar list of differential diagnoses in 3 repetitions, the model’s response is considered consistent. These 5 cases were no longer used in the formal evaluation dataset.

The response of each case was independently evaluated by two senior emergency physicians (SP) (MXM and XKL), who had 5 and 8 years of clinical experience, respectively, following a double-blind assessment protocol. In cases where discrepancies or significant score differences emerged for the same question (e.g., a score of 5 vs. 2), a full discussion was conducted with a third physician (LQ) with at least 15 years of clinical experience. The majority consensus method was then adopted to determine the final score.

### 2.6 Statistical analysis

First, the normality of the data distribution was evaluated through the Shapiro–Wilk test, and then the scoring results of the reports generated by LLMs and EDJP were compared. Since the data showed a non-normal distribution, the Friedman test was used to analyze the overall differences between the main diagnostic accuracy score distributions of 5 LLMs and the EDJP scores. Likewise, *post-hoc* analyses were conducted using pairwise with Bonferroni correction for multiple comparisons to control the familywise error rate (*α* = 0.05/number of comparisons). The age distribution and the comprehensiveness scores of differential diagnoses were described by the median and interquartile range (IQR). The ICC was used to test the scoring consistency of the two reviewers. All data were statistically analyzed using Prism (version 10.0; GraphPad) and SPSS 27.0, and *p* < 0.05 was considered statistically significant.

## 3 Results

### 3.1 Baseline characteristics of cases

**Figure 1:**
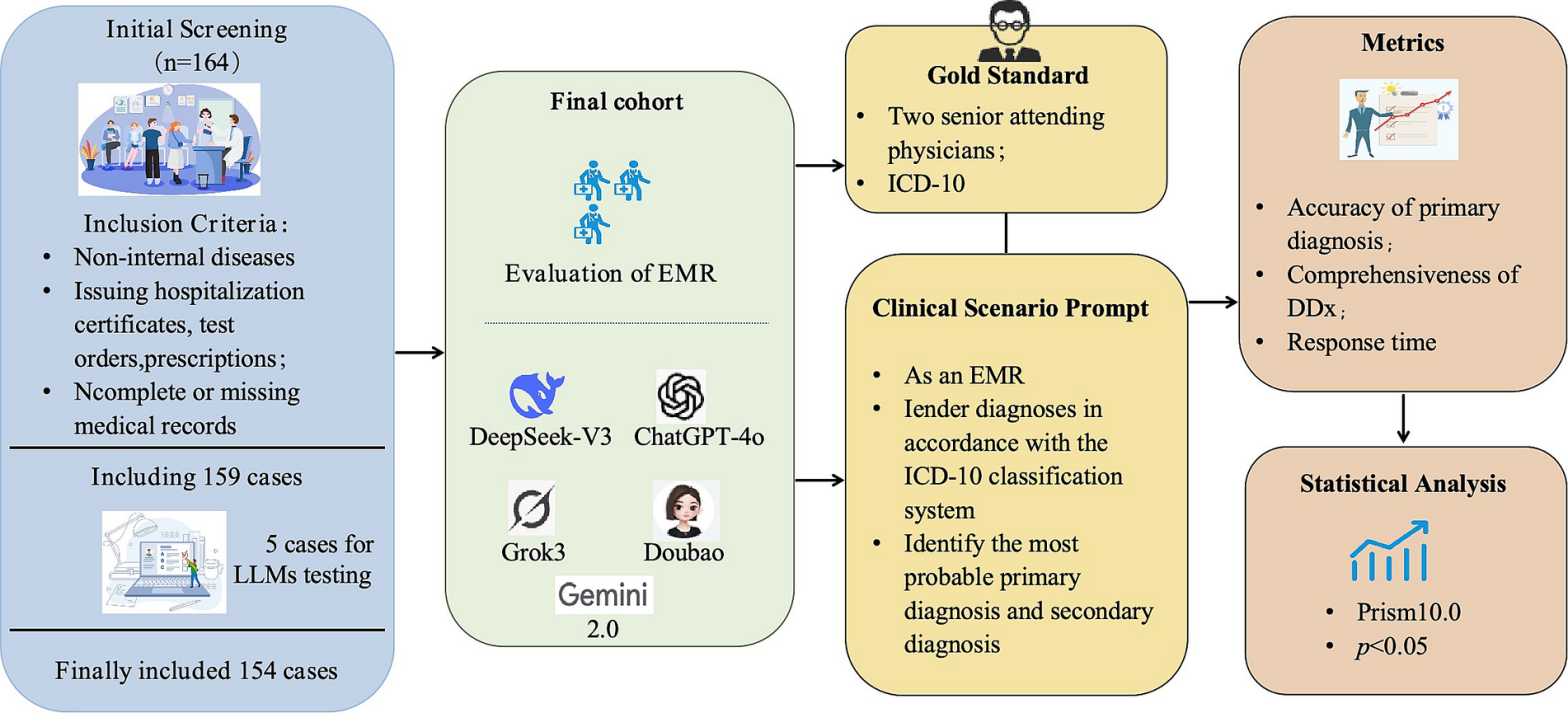
Study design flowchart.

A total of 154 inpatients in the emergency internal medicine department were included in this study. The median age was 45 years (IQR: 31.0-62.0), among which 80 were male (51.9%) and 74 were female (48.1%). The distribution of disease systems was as follows: 33 cases (21.4%) of circulatory system diseases, with a median age of 60.5 years (IQR: 45.0-71.0); 34 cases (22.1%) of respiratory system diseases, with a median age of 44.5 years (IQR: 30.0-60.0); 26 cases (16.9%) of digestive system diseases, with a median age of 37.5 years (IQR: 28.5-49.3); 11 cases (7.1%) of urinary system diseases, with a median age of 56.0 years (IQR: 32.0-65.0); 22 cases (14.3%) of nervous system diseases, with a median age of 55.0 years (IQR: 44.0-65.0); 28 cases (18.2%) of other diseases, with a median age of 33.5 years (IQR: 26.3-49.8) (Table 1). An overview of this study is presented in Figure 1.

**Table 1:** Demographic data (*N* = 154).

Feature	Data
Total number of cases	154
Age	45.0 (31.0-62.0)
Gender (e.g., %)	
Male	80 (51.9)
Female	74 (48.1)
Systemic distribution of diseases (example, %)	
Circulatory system	33 (21.4)
Respiratory system	34 (22.1)
Digestive system	26 (16.9)
Urinary system	11 (7.1)
Nervous system	22 (14.3)
Others	28 (18.2)

### 3.2 Primary diagnostic accuracy

#### 3.2.1 Comparison of primary diagnostic accuracy

Friedman test indicated a statistically significant difference in the final scores reflecting primary diagnostic accuracy between EDPJ and the 5 LLMs (GPT-4o, Gemini-2.0, Grok3, DeepSeek-V3, and Doubao) (*p* < 0.05).

**Figure 2:**
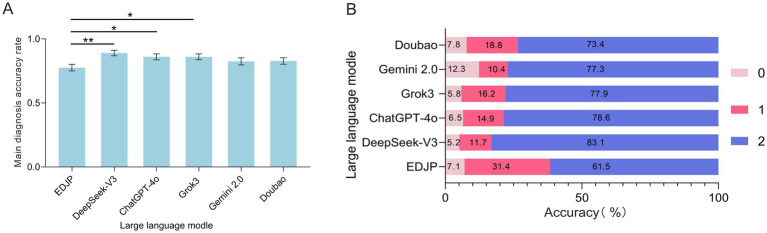
Comparison of diagnostic accuracy of all cases between EDJP and LLMs (DeepSeek-V3, ChatGPT-4o, Grok3, Gemini 2.0, Doubao). **(A)** The bar chart shows the Likert scale (0–2 points) scores of the main diagnostic accuracy of all cases by each subject. The error bars represent the 95% confidence interval, and the scores represent the differences in main diagnostic accuracy. **(B)** The stacked bar chart shows the proportion (%) of the main diagnoses of each subject with different scores of 0, 1, and 2 points. The numbers within the grading segments mark the proportion of scores in this category, presenting the score distribution. *p* values were calculated using Friedman test, with Bonferroni-corrected pairwise comparisons. *: *p*<0.05; **: *p*<0.01.

The results showed that the diagnostic accuracy of DeepSeek-V3, ChatGPT-4o, and Grok3 was significantly higher than that of EDJP (*p* < 0.05, Bonferroni corrected). DeepSeek-V3 had the highest diagnostic accuracy rate, reaching 90.0% (95% CI: 0.85–0.93); followed by Grok3 and ChatGPT-4o, both with an accuracy rate of 86.0% (95% CI: 0.81–0.91 and 0.81–0.91); Gemini-2.0 had an accuracy rate of 86.0% (95% CI: 0.82–0.91); Doubao had an accuracy rate of 82.5% (95% CI: 0.78–0.88); and EDJP had the lowest accuracy rate, at 77.50% (95% CI: 0.73–0.78) (Figure 2A).

Further analysis showed that there was no significant statistical difference in the diagnostic accuracy between Doubao (83.0, 95% CI: 0.78–0.88) and Gemini-2.0 (82.5, 95% CI: 0.77–0.88) compared to EDJP (*p* > 0.05, Bonferroni corrected), and there was no significant statistical difference in the main diagnostic accuracy among the models (*p* > 0.05, Bonferroni corrected) (Figure 2A).

From the perspective of the score distribution, Figure 2B shows that DeepSeek-V3 has the highest main diagnostic accuracy score (2.0 points, 83.1%), which is significantly higher than that of residents (1.7 points, 61.5%, *p* < 0.05, Bonferroni corrected). ChatGPT-4o ranks second, with a total of 121 cases (78.6%). Among the lowest scores (0 points), Gemini-2.0 has the highest frequency, with 19 cases (12.3%); Doubao ranks second, with a total of 12 cases (7.8%).

#### 3.2.2 Diagnostic accuracy analysis of disease subgroups

To further explore the diagnostic performance of LLMs in different systemic diseases, we conducted a stratified analysis of each disease subgroup. Friedman test indicated a statistically significant difference between EDPJ and the 5 LLMs (*p* < 0.05).

**Figure 3:**
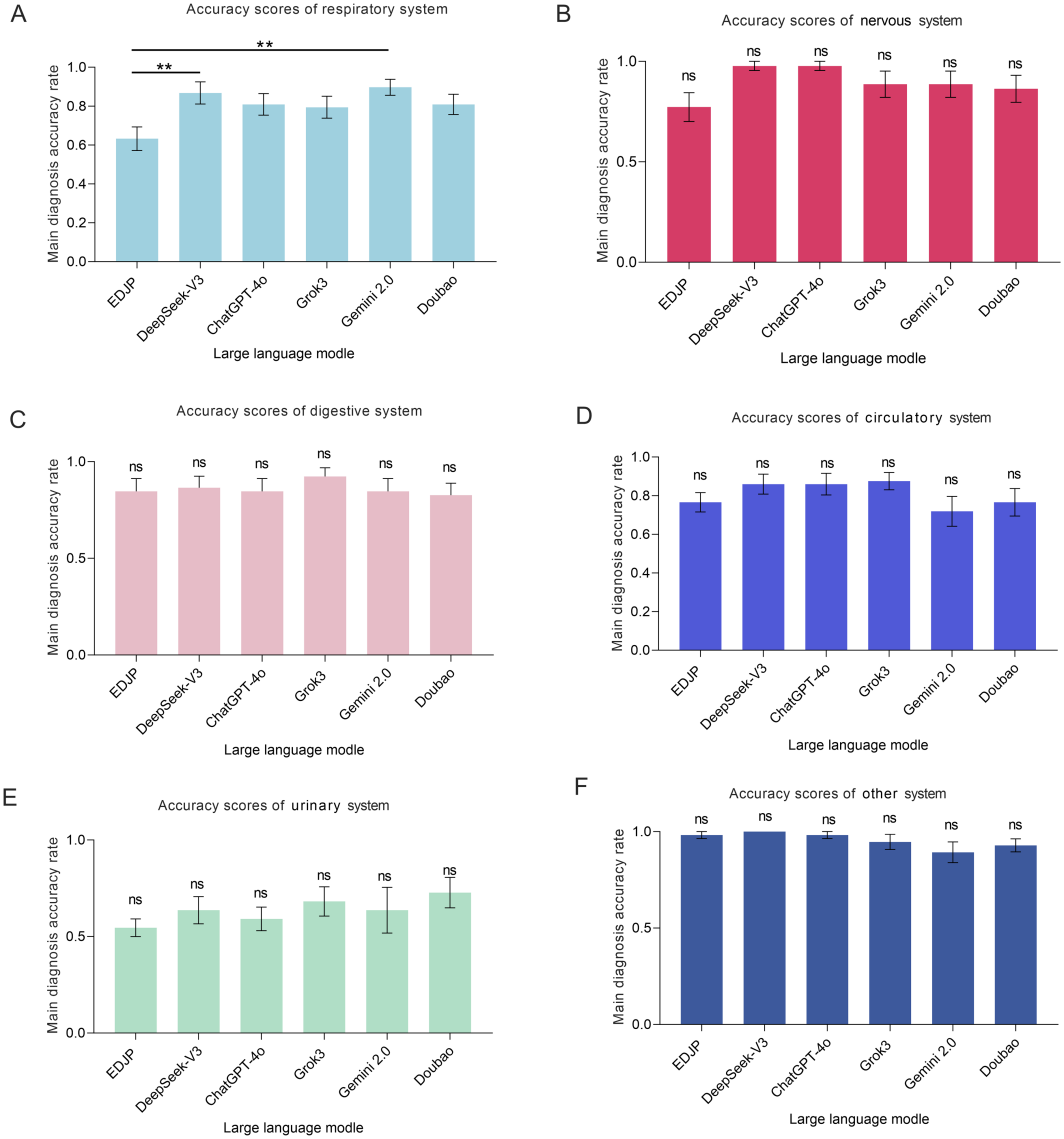
Comparison of the main diagnostic accuracy of subgroup diseases between EDJP and LLMs (DeepSeek-V3, ChatGPT-4o, Grok3, Gemini 2.0, Doubao). **(A)** The bar chart shows the scores and error bars of the main diagnostic accuracy of respiratory system diseases for each subject; **(B)** The bar chart shows the scores and error bars of the main diagnostic accuracy of nervous system diseases for each subject; **(C)** The bar chart shows the scores and error bars of the main diagnostic accuracy of digestive system diseases for each subject; **(D)** The bar chart shows the Likert scale scores and error bars of the main diagnostic accuracy of circulatory system diseases for each subject; **(E)** The bar chart shows the Likert scale scores and error bars of the main diagnostic accuracy of urinary system diseases for each subject; **(F)** The bar chart shows the Likert scale scores and error bars of the main diagnostic accuracy of other system diseases for each subject. The error bars represent the 95% confidence interval, and the scores represent the differences in main diagnostic accuracy. **: *p*<0.01.

For example, respiratory diseases: The diagnostic accuracies of Gemini-2.0 (89.7, 95% CI: 0.81–0.98) and DeepSeek-V3 (86.8, 95% CI: 0.75–0.98) were significantly higher than that of EDJP (63.2, 95% CI: 0.51–0.76) (*p* < 0.05, Bonferroni corrected); there were no statistically significant differences between the remaining models and EDJP, as well as among the LLMs (*p* > 0.05, Bonferroni corrected) (Figure 3, Table 2).

**Table 2:** Comparison of main diagnostic accuracy between LLMs and EDJP in respiratory system.

Models	Accuracy	95%CI	Adjusted *p* value
EDJP	63.2%	(0.51–0.76)	*p* > 0.05^b^
DeepSeek-V3	86.7%	(0.75–0.98)	0.0046^a^
GPT-4o	80.1%	(0.70–0.92)	0.1791^b^
Grok3	79.4%	(0.68-0.91)	0.3405^b^
Gemini-2.0	89.7%	(0.81–0.98)	0.0045^a^
Doubao	80.9%	(0.70-0.91)	0.2755^b^

*p* values were calculated using Chi-square test, with Bonferroni-corrected pairwise comparisons.

a Refers to statistical significance between DeepSeek-V3, Gemini-2.0 and EDJP.

b Refers to comparisons between 5 LLMs and EDJP.

In circulatory, digestive, nervous, urinary and other system diseases, there were no significant differences in diagnostic accuracy between each model and EDJP (*p* > 0.05, Bonferroni corrected), and there were also no statistical differences among the models (Supplementary Table S3).

### 3.3 Comprehensive analysis of differential diagnosis

The comprehensive scores of differential diagnoses for the 5 LLMs were significantly higher than EDJP (*p* < 0.05, Friedman test). Among them, DeepSeek-V3 had the highest median comprehensive score of 5.0 (IQR: 5.0–5.0); Gemini-2.0 and Grok3 also had a median of 5.0 (IQR: 5.0–5.0); ChatGPT-4o and Doubao had a median of 5.0 (IQR: 4.0–5.0); EDJP had the lowest score with a median of 4.0 (IQR: 4.0–5.0).

**Figure 4:**
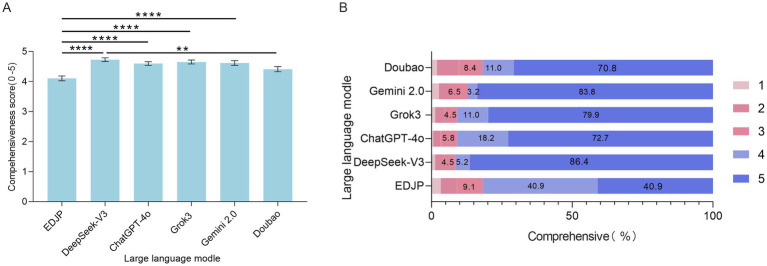
Comprehensive comparison of differential diagnosis between clinicians and LLMs (DeepSeek-V3, ChatGPT-4o, Grok3, Gemini 2.0, Doubao). **(A)** The bar chart shows the Likert scale (1–5 points) scores of each subject on the comprehensiveness of differential diagnosis for all cases. The error bars represent the 95% confidence interval, and the scores reflect the differences in the comprehensiveness of differential diagnosis. **(B)** The stacked bar chart shows the proportion (%) of differential diagnoses of each subject in different scores from 1 to 5 points. The numbers within the grading segments indicate the proportion of scores in this category, presenting the distribution of comprehensiveness scores. **: *p*<0.01; ****: *p*<0.0001.

Inter-model comparisons showed that the comprehensiveness of differential diagnosis of DeepSeek-V3 was significantly better than that of Doubao (*p* < 0.05, Bonferroni corrected); while there was no significant difference between Gemini-2.0, GPT-4o, Grok3 and Doubao (*p* > 0.05, Bonferroni corrected) (Figure 4A).

From the perspective of the rating distribution, Figure 4B shows DeepSeek-V3 has the highest proportion in the highest rating (5 points), with a total of 133 cases, accounting for 86.4%; followed by Gemini-2.0 (129 cases, 83.8%), Grok3 (123 cases, 79.9%), ChatGPT-4o (112 cases, 72.7%), and Doubao (109 cases, 70.8%). In contrast, EDJP has the lowest proportion in the highest rating group, with only 63 cases, accounting for 40.9%. It is worth noting that EDJP has the highest frequency in the 4-point rating group (63 cases, 40.9%), and also has the highest proportion in the lowest rating (1 point) group, with a total of 5 cases, accounting for 3.2%; while DeepSeek-V3 has the lowest proportion in the 1-point rating group, with only 1 case, accounting for 1.3% (Figure 4B).

### 3.4 Response time analysis

The response times of the 5 LLMs were significantly faster than that of EDJP (*p* < 0.0001, Friedman test). Specifically, Doubao had the shortest response time of 6.3 s (IQR: 5.3–7.9); followed by Gemini-2.0 (6.4 s, IQR: 5.7–7.0), Grok3 (7.1 s, IQR: 6.6–7.5), ChatGPT-4o (9.5 s, IQR: 8.5–10.5); DeepSeek-V3 had the longest response time of 14.0 s (IQR: 10.6–15.9); and the response time of EDJP was 360.2 s (IQR: 289.0–400.0).

**Figure 5:**
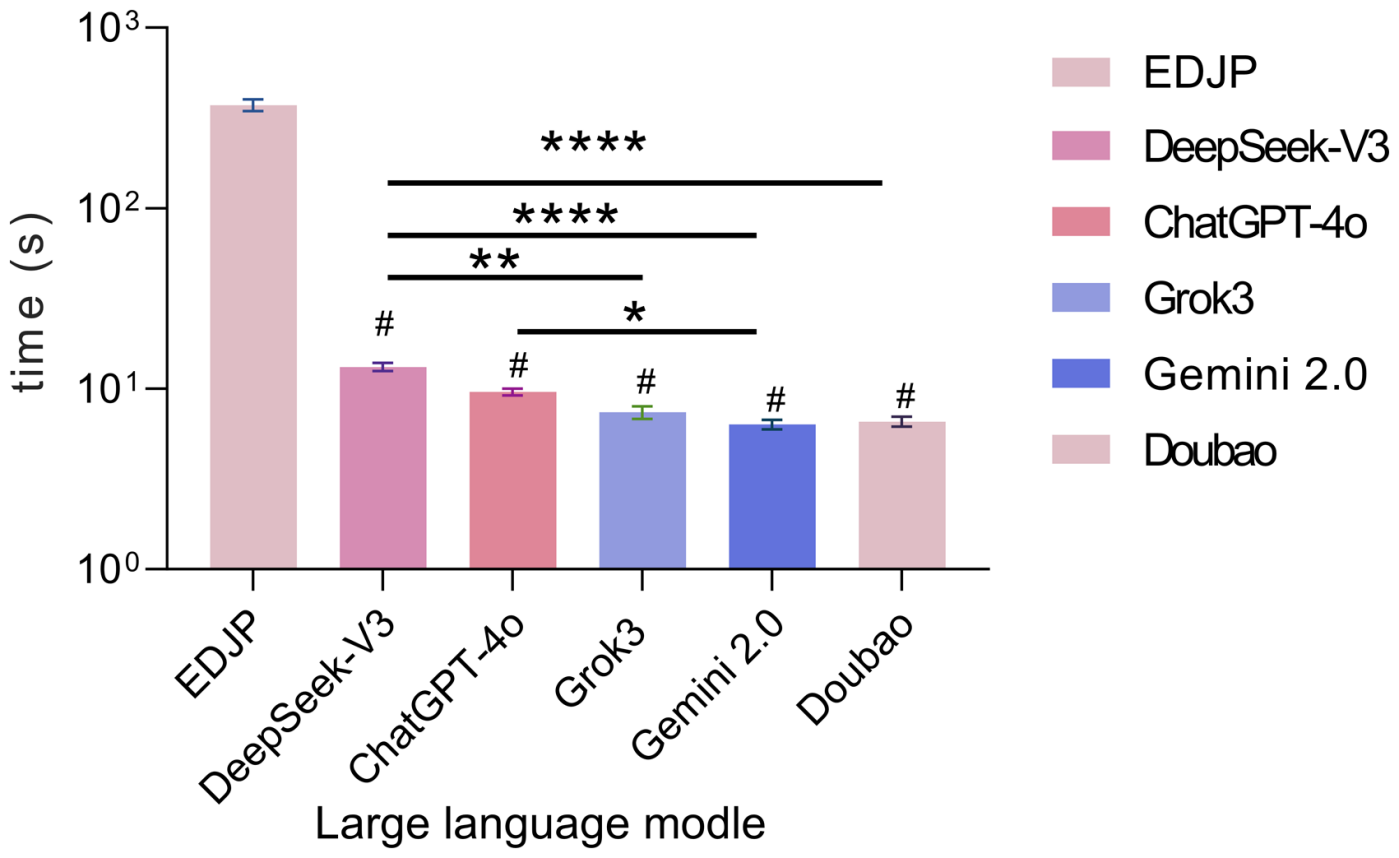
Comparison of response times between EDJP and LLMs (DeepSeek-V3, ChatGPT-4o, Grok3, Gemini 2.0, Doubao). The bar chart shows the response time (seconds), and the error bars represent the 95% confidence interval, which is used to compare the response speeds of different entities. ^#^Indicates that the difference in comparison between 5 LLMs and EDJP is statistically significant (*p* < 0.05). *: *p*<0.05; **: *p*<0.01; ****: *p*<0.0001.

Comparisons among the models showed that the response times of Doubao, Gemini-2.0, and Grok3 were significantly faster than that of DeepSeek-V3 (*p* < 0.05, Bonferroni corrected); the response time of Gemini-2.0 was significantly faster than that of ChatGPT-4o (*p* < 0.05, Bonferroni corrected); there was no significant difference in response times among the remaining models (*p* > 0.05, Bonferroni corrected) (Figure 5).

### 3.5 Inter-rater reliability

Two emergency SP showed excellent consistency in judging the accuracy of the primary diagnosis results (ICC, 0.899; 95% CI: 0.864–0.926); they also showed moderate consistency in the scoring interpretation of the comprehensiveness of differential diagnoses (ICC, 0.617; 95% CI: 0.565–0.664).

## 4 Discussion

This retrospective analysis evaluated 5 LLMs’ diagnostic performance against EDJPs in real emergency internal medicine settings. Results showed DeepSeek-V3, ChatGPT-4o, and Grok3 achieved significantly higher primary diagnostic accuracy. All LLMs outperformed EDJPs in differential diagnosis comprehensiveness, with faster response times-Doubao being the quickest. These findings highlight LLMs’ potential in enhancing emergency diagnosis through improved accuracy, comprehensiveness, and efficiency. DeepSeek-V3, ChatGPT-4o, and Grok3’s strong performance in real emergencies may stem from optimized reasoning models, multimodal integration, and causal logic generation. Urda-Cîmpean et al. (8) found DeepSeek-V3 superior across evaluation dimensions, particularly in medical reasoning, with all models excelling more in knowledge-based than reasoning tasks. While Hoppe et al. (19) reported GPT-4’s 90% diagnostic accuracy in 100 emergency cases, LLM accuracy remains controversial. Shan et al. (20) noted wide fluctuations (25–97.8%) versus clinician accuracy, with most studies at high bias risk. Chan et al. showed DeepSeek-R1 and GPT-4 had 35 and 39% final diagnostic accuracy but low correct differential diagnosis inclusion (48% vs. 64%), indicating complex reasoning limits.

This study confirmed all 5 LLMs outperformed EDJPs in differential comprehensiveness, aligning with prior research (21). DeepSeek-V3 led in accuracy, comprehensiveness, and highest scores, leveraging its ability to integrate vast medical data for systematic diagnoses. Wu et al. (21) demonstrated DeepSeek-R1’s 60% accuracy in top-score groups (vs 27% for non-AI-assisted physicians), with AI-assisted physicians reaching 58% (22). Patel et al. (22) found ChatGPT-4o, Gemini 2.0, and DeepSeek’s average differential scores (87.5%) comparable to clinicians (81.3–96.9%). DeepSeek-V3’s excellence may relate to innovative training (e.g., reinforcement learning pre-training) and architecture, as Zhou et al. (23). noted its superiority in spinal surgery patient education.

However, LLMs face challenges: interpretability, workflow integration, and diverse population validation, with limited differential diagnosis impact evaluations. Missed key medical histories/signs caused LLM diagnostic errors, mirroring human errors (23). Gemini-2.0 had the lowest scores (19 times, 12.34%), linked to its smaller-capacity Pathways architecture—general capabilities not translating to specialized expertise (24, 25). In radiology assessments, ChatGPT outperformed Gemini in accuracy (chest radiology: 100% vs. 83.33%; ultrasound: 100% vs. 63.64%) (26, 27).

EDJPs showed lower overall accuracy but double the 1-point score rate (31.4%), often due to vague diagnoses or guideline over-reliance (e.g., “respiratory tract infection” vs. LLMs’ “acute suppurative tonsillitis”). EDJPs also had lower secondary diagnosis coverage, while LLMs automatically included relevant ones via test/history analysis. All models and EDJPs performed poorly in urinary system diseases, with top-performing Doubao at 72.7% accuracy. This may be related to the characteristics of the model-as general LLMs, their performance may be limited by the coverage and pertinence of the training data.

Although LLMs perform excellently in the comprehensive score of differential diagnosis, the ‘redundancy problem’ of over—generating irrelevant diagnoses may undermine their clinical utility (28, 29). For example, in the pre-experimental stage of this study, by instructing to limit the primary diagnosis to no more than 2 and the secondary diagnosis to no more than 5, LLMs often misinterpreted it as the “target” quantity and generated unnecessary diagnoses to “make up the numbers” (Supplementary Table S4) LLMs often generate unnecessary or minimally relevant diagnoses just to “make up the numbers”. It may lead to unnecessary examinations, increase patient anxiety and may distract doctors from truly high-risk diagnoses. For instance, for a case of “upper respiratory tract infection”, listing “lung cancer” and “systemic lupus erythematosus” as secondary diagnoses, while “correct” from the perspective of “comprehensiveness”, is completely unacceptable from the perspectives of clinical practicality and patient safety. This reveals the limitations of the current evaluation framework: we only employed a “comprehensiveness” metric to reward such behavior, yet lacked corresponding “precision” or “clinical relevance” metrics for penalization. Moreover, the 5-point Likert scale merely emphasizes the inclusion of reasonable diagnoses, with no penalty mechanism for irrelevant or low-probability diagnoses, thus granting positive scores to redundant diagnoses. This may lead to artificially inflated scores for LLMs, rendering their apparent accuracy higher than that of the physician group.

Future research and model development must take generating high-quality and highly relevant diagnostic lists as the core objective, rather than simply pursuing “quantity.” Without strict clinical supervision, such errors may lead to misdiagnosis or adverse events. Therefore, all results generated by LLMs currently should be strictly regarded as reference information and must be used under close clinical supervision (30).

In response time, LLMs outperform EDJP significantly due to efficient algorithms and robust computing power. In this study, we found that Doubao is fastest while DeepSeek-V3 is slowest, though the latter achieves higher accuracy-likely tied to model architecture, requiring scenario-specific trade-offs. Wu et al.’s (21) prospective study demonstrated LLM assistance reduced EDJPs’ median diagnostic time from 1920s to 972 s. This finding is consistent with the core conclusion of our study. Notably, we only simulated and compared the response time of LLMs and EDJPs in independent diagnostic tasks, and did not evaluate the impact of LLM clinical workflow integration on physicians’ real-world decision-making. The rapid response of LLMs offers potential for emergency triage and preliminary diagnosis assistance, but this clinical value needs further verification via prospective real-world studies. This study innovates LLM clinical evaluation by using real critical emergency cases, capturing the ED’s “complex, chaotic, urgent” nature-unlike prior research on standardized/simulated cases (12, 21, 24).

This study has certain limitations. Firstly, this study has a single-center retrospective design. The cases are regional. Although the 154 patients included cover multiple systemic diseases, the sample size is limited, which may affect the representativeness and universality of the results. Noting the unbalanced sample size across disease system subgroups, especially the small sample size of urinary system diseases (11 cases, 7.1%), which leads to insufficient statistical power and limited generalizability of the results for this subgroup. Secondly, the 15-emergency department EDJP participating in the comparison all have 3–5 years of work experience, and they cannot fully represent the overall level of EDJP, restricting the extensive extrapolation of the differences in diagnostic capabilities between LLMs and human doctors. Third, only 5 cases were included in the stability test of this study, accounting for 3.2% of the total cases. This sample size is insufficient to fully verify the stability of LLMs in the context of the present study.

To enhance the practicality and reliability of LLMs in emergency diagnosis, we offer the following insights. First, integrate multimodal data-combining text with imaging, lab tests, and real-time vital signs to build comprehensive decision-support frameworks for critically ill patients. Second, develop emergency-specific LLMs tailored to critical subtypes like trauma and sepsis. Finally, clarify clinical integration principles, shifting from “human replacement” to effective human-machine collaboration. Against this background, we propose a novel accuracy-oriented evaluation scheme that penalizes irrelevant or low-probability diagnoses:(1)Primary diagnosis: Top-1 diagnostic accuracy (%); (2) Secondary diagnoses: Top-*n* accuracy (*n* = 1–10), defined as the proportion of cases where the correct diagnosis is included in the top n differential diagnoses; (3) Penalty mechanism: 1 point is deducted for every additional 10% of irrelevant/low-probability diagnoses (1 point for 10%, 2 points for 20%, 3 points for 30%, 4 points for 40%, 5 points for ≥50%); incorrect diagnoses receive 0 points.

## 5 Conclusion

In the real-world emergency internal medicine scenarios, LLMs exhibit overall diagnostic performance superior to that of the emergency EDJP. Specifically, the main diagnostic accuracies of DeepSeek-V3, ChatGPT-4o, and Grok3 are better than EDJP. In the subgroup of respiratory diseases, Gemini-2.0 and DeepSeek-V3 perform even better. The comprehensiveness of differential diagnosis of all LLMs is significantly better than that of EDJP, and their response speed is faster. Doubao has the fastest response speed among them. The above results indicate that LLMs have significant application potential in clinical decision-support among EDJP. With further optimization aligned with clinical needs, human-machine collaboration is expected to enhance diagnostic efficiency and accuracy, promoting broader LLM application in emergency.

## Data Availability

The raw data supporting the conclusions of this article will be made available by the authors, without undue reservation.
