# Supplementary material for: Performance evaluation of large language models in the diagnosis of emergency internal medicine diseases: a retrospective study
Source: Front Public Health. 2026 May 8;14:1780425. doi: 10.3389/fpubh.2026.1780425 (PMC13195009; doi:10.3389/fpubh.2026.1780425)
Supplement: Supplementary file 1 [file Table_1.DOCX]

**Case 1.**

**1.Basic Information：**Patient：xx, male, 49 years old,.

**2.Chief complaint:** Abdominal pain for more than 1 week.

**3.Present illness:** The patient reported that more than a week ago, they experienced distending pain and discomfort in the middle and upper abdomen without obvious inducement, without nausea or vomiting, without fever, and without chest tightness or shortness of breath, so they came to our hospital for treatment.

**4.History:** No allergies; no history of diseases; medication history includes rivaroxaban tablets; 10 years ago, he underwent mechanical valve Bentall + total arch replacement + descending aorta intraoperative stent surgery in our hospital for aortic root aneurysm and type A aortic dissection; no history of trauma, family history, etc.

**5.Marital and childbearing history:** Married.

**6.Physical examination:** Vital signs: Blood pressure 121/74mmHg, body temperature (ear temperature) 37℃, pulse 112 beats/min, respiratory rate 14 breaths/min, oxygen saturation 97%, heart rate 112 beats/min, height 175cm, weight 65kg, high-risk fall assessment as high risk.

**7.Examination:** Clear consciousness, good mental state, no deformity in the appearance of the skull, bilateral pupils equal in size and round, sensitive to light reflex, soft neck without resistance, breath sounds in both lungs slightly rough, no obvious dry or wet rales heard, regular heart rhythm, no obvious pathological murmurs heard, abdomen slightly distended, mild tenderness in the mid-upper abdomen, no rebound tenderness, no obvious abnormalities in the rest of the abdomen, normal movement of the spine and limbs.

**7.1 Examination results**

Chest high-resolution CT plain scan, 2025-05-03 01:14 **Examination description:** Emergency chest CT, clinically indicating abdominal pain. Imaging shows exudation and atelectasis in the left lower lung; no obvious tracheal or bronchial foreign bodies; no obvious esophageal foreign bodies; no signs of pneumothorax or hydropneumothorax.

**7.2 CT aortography (TAVI),** 2025-05-03 01:14 **Examination description:** Imaging shows postoperative aortic dissection, with surrounding encapsulation and enhancement of the ascending aorta - aortic arch, considered as pseudoaneurysm with local organization, intimal dissection from the thoracic aorta to the level of the right common iliac artery, forming true and false lumens, with the false lumen larger than the true lumen, significant widening of the false lumen in the lower segment of the abdominal aorta, and contrast agent opacification in the false lumen. The celiac trunk, superior mesenteric artery, and left renal artery originate from the true lumen, the right renal artery and inferior mesenteric artery open into the false lumen. Multiple exudations, blood accumulation, and flaky contrast agent are visible around the abdominal aorta.**Examination report:** Postoperative aortic dissection, with surrounding encapsulation and enhancement of the ascending aorta - aortic arch, considered as pseudoaneurysm with local organization, widening of the abdominal aortic false lumen, obvious exudation, blood accumulation, and visible contrast agent around the corresponding segment of the abdominal aorta, suggesting possible rupture.

**8.Laboratory results**

8.1 Blood Routine Related Indicators

|1 | White Blood Cell Count | 17.9↑|4.0 - 10.0×10⁹/L | Mindray Flow Cytometry |

|2 | Red Blood Cell Count | 4.29|4.09 - 5.74×10¹²/L | Mindray Sheath Flow Method |

|3 | Hemoglobin | 114↓|131 - 172g/L | Mindray Colorimetric Method |

|4 | Hematocrit | 0.336↓|0.380 - 0.508L/L | Calculation Method |

|5 | Mean Corpuscular Volume | 78.3↓|84.0 - 94.0fL | Calculation Method |

|6 | Mean Corpuscular Hemoglobin | 26.5|27.0 - 34.0pg | Calculation Method |

|7 | Mean Corpuscular Hemoglobin Concentration | 339|320 - 380g/L | Calculation Method |

|8 | Red Blood Cell Distribution Width | 17.3↑|12.0 - 15.0%| Calculation Method |

|9 | Platelet Count | 332|100 - 300×10⁹/L | Mindray Sheath Flow Method |

|10 | Plateletcrit | 0.292|0.106 - 0.25%| Mindray Sheath Flow Method |

|11 | Mean Platelet Volume | 8.8|7.8 - 11.3fL | Calculation Method |

|12 | Absolute Neutrophil Count | 15.33↑|2.00 - 7.00×10⁹/L | Calculation Method |

|13 | Absolute Lymphocyte Count | 1.56|0.80 - 4.00×10⁹/L | Calculation Method |

|14 | Absolute Monocyte Count | 0.89|0.20 - 1.20×10⁹/L

|15 | Absolute value of eosinophils | 0.09| <1.00×10⁹/L | Calculation method |

|16 | Absolute value of basophils | 0.02| <0.10×10⁹/L | Calculation method |

|17 | Neutrophil percentage | 85.7↑|50.0 - 70.0%| Mindray flow fluorescence |

|18 | Lymphocyte percentage | 8.7↓|20.0 - 40.0%| Mindray flow fluorescence |

|19 | Monocyte percentage | 5.0|4.0 - 12.0%| Mindray flow fluorescence |

|20 | Eosinophil percentage | 0.5| <10.0%| Mindray flow fluorescence |

|21 | Basophil percentage | 0.1| <1.0%| Mindray flow fluorescence |

|22 | Whole blood CRP|180.2↑| <10.0mg/L | Mindray immunoturbidimetry |

**8.2 Blood Biochemical Related Indicators**

| Serial Number | Item Name | Test Result | Reference Range | Unit | Test Method |

|1 | Total Bilirubin | 6.6| <26.0μmol/L | Siemens Oxidase Method |

|2 | Direct Bilirubin | 1.8| <4.0μmol/L | Siemens Oxidase Method |

|3 | Indirect Bilirubin | 4.8|5.0 - 20.0μmol/L | Calculation Method |

|4 | Total Protein | 75.9↓|66.0 - 83.0g/L | Siemens Biuret Method |

|5 | Albumin | 31.3|35.0 - 52.0g/L | Siemens Bromocresol Green Method |

|6 | Globulin | 44.6|15.0 - 30.0g/L | Calculation Method |

|7 | Albumin / Globulin Ratio | 0.70|1.20 - 2.40 | Calculation Method |

|8 | Alanine Aminotransferase | 9|9 - 50U/L | Siemens Rate Method |

|9 | Creatine Kinase | 346↑| <164U/L

|10 | Creatine Kinase - MB|<1| ≤5ng/mL | Ruiyuan Turbidimetric Quality Method |

|11 | Aspartate Aminotransferase | 21|15 - 40U/L | Siemens Enzymatic Method |

|12 | Lactate Dehydrogenase | 346|120 - 250U/L | Siemens Rate Method |

|13 | Troponin - I|0.014| <0.053ng/mL | Siemens Chemiluminescence Method |

|14 | Potassium | 3.41↓|3.50 - 5.30mmol/L | Siemens Ion - Selective Electrode Method |

|15 | Sodium | 133.7↓|137.0 - 147.0mmol/L | Siemens Ion - Selective Electrode Method |

|16 | Chloride | 102.4|99.0 - 110.0mmol/L | Siemens Ion - Selective Electrode Method |

|17 | Calcium | 2.01↓|2.11 - 2.52mmol/L | Siemens Colorimetric Method |

|18 | Glucose | 5.66|3.89 - 6.11mmol/L

|19 | Urea | 3.63|2.80 - 7.60mmol/L | Siemens Urease Method |

**8.3 Coagulation function-related indicators**

| Serial Number | Item Name | Test Result | Reference Range | Unit | Test Method |

| 1 | Prothrombin Time | 19.6↑ | 12.0 - 14.0 seconds | stago coagulation method |

| 2 | Prothrombin Time Activity | 48.0↓ | 90.0 - 137.0% | Calculation method |

| 3 | International Normalized Ratio | 1.65↑ | 0.9 - 1.1 | Calculation method |

| 4 | Activated Partial Thromboplastin Time | 58.6 | 30.0 - 45.0 seconds | stago coagulation method |

| 5 | Thrombin Time | 18.7 | <20.0 seconds | stago coagulation method |

| 6 | Plasma Fibrinogen | 8.03↑ | 2.0 - 4.0g/L | stago coagulation method |

| 7 | D - Dimer | 3770↑ | <500μg/L (FEI) | stago immunoturbidimetry |

**8.4 Blood Gas Analysis Related Indicators**

| Serial Number | Item Name | Test Result | Reference Range | Unit | Test Method |

| 1 | Blood pH | 7.459↑ | 7.350 - 7.450 | | Electrode Method |

| 2 | Partial Pressure of Oxygen | 98.6↑ | 75.0 - 95.0 mmHg | | Electrode Method |

| 3 | Partial Pressure of Carbon Dioxide | 35.6↓ | 36.0 - 44.0 mmHg | | Electrode Method |

| 4 | Corrected Blood pH | 7.459 | 7.350 - 7.450 | | Calculation Method |

| 5 | Whole Blood Base Excess | 2.9 | -3.0 - 3.0 mmol/L | | Calculated Value |

| 6 | Actual Bicarbonate Concentration | 24.9 | 22.0 - 26.0 mmol/L | | Calculated Value |

| 7 | Standard Bicarbonate Concentration | 25.9 | 22.0 - 26.0 mmol/L| |Calculated Value |

| 8 | Anion Gap | 6.6↓ | 8.0 - 16.0 mmol/L | | Calculation Method |

| 9 | Corrected Partial Pressure of Oxygen | 98.6 | 75.0 - 95.0 mmHg | | Calculation Method |

| 10 | Blood Oxygen Concentration | 15.9 | 15.0 - 22.0 Vol%| |Calculated Value |

| 11 | Oxygen Saturation | 98.1↑ | 95.0 - 98.0%| |Calculated Value |

| 12 | Oxyhemoglobin Percentage | 97.2↑ | 94.0 - 97.0%| |Optical Method |

| 13 | Deoxyhemoglobin Percentage | 1.9 | < 5.0%| |Optical Method |

| 14 | Methemoglobin Percentage | 0.5 | < 1.5%| |Optical Method |

| 15 | Carboxyhemoglobin Percentage | 0.4 | 0.5 - 2.0%| |Optical Method |

| 16 | Corrected Partial Pressure of Carbon Dioxide | 35.6 | 36.0 - 44.0 mmHg | | Calculation Method

| 17 | Total Carbon Dioxide | 26.0 | 23.0 - 27.0 mmol/L| |Calculated Value |

| 18 | Body Temperature | 37.0 | 36.5 - 37.5 ℃| |Input Value |

| 19 | Whole Blood Lactic Acid | 0.90 | 0.50 - 1.60 mmol/L| |Electrode Method |

| 20 | Glucose | 5.9 | 3.89 - 6.11 mmol/L| |Electrode Method |

| 21 | Potassium (K+) | 3.40↓ | 3.50 - 5.30 mmol/L| |Electrode Method |

| 22 | Sodium (Na+) | 137.0 | 137.0 - 147.0 mmol/L

|23 | Chloride (Cl ^-^) |105.0|99.0 - 110.0mmol/L | Electrode method |

|24 | Ionized calcium | 1.07↓|1.15 - 1.29mmol/L | Electrode method |
**promopt：**

I am an emergency department physician managing an emergency patient. Based on the provided clinical data (history, physical examination, tests, imaging). Please make a diagnosis according to the ICD-10 disease classification, make the most likely diagnosis, and divide it into the main diagnosis and secondary diagnosis (no explanation required).

**Case 2:**
**1.Basic Information：**Patient xx, female, 71 years old.

**2. Chief complaint:** Low back soreness for 1 week, difficulty moving both lower limbs for 3 days

**3. Present illness history:** The patient experienced low back soreness and discomfort without obvious inducement 1 week ago, accompanied by palpitations, no dizziness or headache, accompanied by dysuria, no obvious gross hematuria, no chest tightness or shortness of breath, no abdominal pain or bloating, and no special treatment was given. About 3 days ago, the patient had difficulty moving both lower limbs and was unable to walk. She went to a local hospital for relevant examinations and symptomatic treatment, but there was no obvious improvement. Now she comes to our hospital.

**4. History:** No history of allergies, has hypertension for 20 years, medication history is nifedipine, no history of surgery, no history of trauma, no family history.

Marital and childbearing history: Married and has given birth, not pregnant, postmenopausal.

Physical examination:

**5. Vital signs:** Blood pressure 152/76 mmHg, body temperature (ear temperature) 37℃, pulse 107 beats/min, respiratory rate 4 breaths/min, oxygen saturation 92%, heart rate 107 beats/min, height 161 cm, weight 72 kg, high-risk fall assessment is high-risk.

**6.Physical examination content:** Clear consciousness, listless spirit, no deformity of the skull, bilateral pupils are equal in size and round, with a diameter of about 3 mm, and bilateral light reflexes are sensitive. Auscultation shows rough breath sounds in both lungs, no obvious dry and wet rales in both lungs. Heart sounds are strong, rhythm is regular, no heart murmur is heard. Abdomen is flat and soft, no tenderness or rebound tenderness in the entire abdomen, no percussion pain in the liver and kidney areas. Muscle strength and muscle tone of the limbs are normal, and pathological signs are not elicited.

**7.Examination results**
**7.1 Chest High-Resolution CT Plain Scan: 2025 - 05 - 01 15:37, Examination Description:** Emergency chest CT, clinically suggestive of infection. Imaging shows esophageal intubation status; bilateral pulmonary exudation, partial consolidation, thickening of interlobular septa, a small amount of bilateral pleural effusion with adjacent pulmonary atelectasis. No obvious tracheal or bronchial foreign bodies; no signs of pneumothorax or hydropneumothorax; no obvious mediastinal emphysema or space-occupying lesions.**Examination Report:** Bilateral pulmonary exudation, partial consolidation, thickening of interlobular septa, a small amount of bilateral pleural effusion with adjacent pulmonary atelectasis, re-examination is recommended; dilatation of the main pulmonary artery and ascending aorta, and a full heart; calcification of the aorta and coronary arteries.

**7.2 CT Pulmonary Angiography: 2025 - 05 - 02 10:19 Examination Description:** Emergency pulmonary artery CTA, clinically suggestive of low back pain for 1 week and inability to move both lower limbs for 3 days. Imaging of pulmonary artery CTA shows a small filling defect in the local branch artery of the left lower lobe; the remaining main pulmonary artery, and the major branches in the left and right lungs are clearly visualized, with no obvious luminal dilation or stenosis, and no obvious filling defects. Incidental findings: gastric tube indwelling; multiple bilateral pulmonary exudations, partial with consolidation, thickening of interlobular septa, a small amount of bilateral pleural effusion with adjacent pulmonary atelectasis. Dilatation of the main pulmonary artery enlarged heart, calcification of the aorta and coronary arteries. **Examination Report:** A small filling defect in the local branch artery of the left lower lobe, considered as embolism.

**7.3Hepatobiliary Pancreatic Splenic Color Doppler Ultrasound Examination: 2025 - 05 - 03 10:58 Examination Description:** Bedside ultrasound. The liver is normal in size, with a smooth capsule, fine parenchymal echoes, good echoes, and CDFI shows unobstructed blood flow. The gallbladder is enlarged, about 8.6×4.6 cm in size, with a smooth wall, and Doppler examination shows no abnormal blood flow. A fluid dark area is seen in the gallbladder fossa. The pancreas and spleen are normal in size and shape, with uniform parenchymal echoes and no abnormalities. **Examination Report:** Fatty liver; possible cholecystitis; sludge in the gallbladder; no obvious abnormalities in the pancreas and spleen; no obvious abnormalities in both kidneys and bilateral ureters.
**8. Test Results**

**8.1Blood Routine Related Indicators**

| Serial Number | Item Name | Result | Reference Range | Unit |

| 1 | White Blood Cell Count | 17.9↑| 4.0 - 10.0×10⁹/L|

| 2 | Red Blood Cell Count | 4.13| 3.68 - 5.13×10¹²/L|

| 3 | Hemoglobin | 130| 113 - 151g/L|

| 4 | Hematocrit | 0.379| 0.335 - 0.450L/L|

| 5 | Mean Red Blood Cell Volume | 91.7| 84.0 - 94.0fL|

| 6 | Mean Red Blood Cell Hemoglobin Content | 31.4| 27.0 - 34.0pg|

| 7 | Mean Red Blood Cell Hemoglobin Concentration | 343| 320 - 380g/L|

| 8 | Red Blood Cell Distribution Width | 13.1| 12.0 - 15.0%|

| 9 | Platelet Count | 51↓| 100 - 300×10⁹/L|

| 10 | Platelet Hematocrit | 0.058↓| 0.106 - 0.25%|

| 11 | Mean Platelet Volume | 11.4↑| 7.8 - 11.3fL|

| 12 | Absolute Neutrophil Count | 16.18↑| 2.00 - 7.00×10⁹/L|

| 13 | Absolute Lymphocyte Count | 1.06| 0.80 - 4.00×10⁹/L|

| 14 | Absolute Monocyte Count | 0.63| 0.20 - 1.20×10⁹/L

| 15 | Absolute value of eosinophils | 0.07 | <1.00×10⁹/L |

| 16 | Absolute value of basophils | 0.00 | <0.10×10⁹/L |

| 17 | Neutrophil percentage | 90.2↑ | 50.0 - 70.0% |

| 18 | Lymphocyte percentage | 5.9↓ | 20.0 - 40.0% |

| 19 | Monocyte percentage | 3.5↓ | 4.0 - 12.0% |

| 20 | Eosinophil percentage | 0.4 | <10.0% |

| 21 | Basophil percentage | 0.0 | <1.0% |

**8.2 Indicators related to urine tests**

| Serial Number | Item Name | Result | Reference Range | Unit |

| 1 | Urine color | Yellow | Light yellow | - |

| 2 | Turbidity | Clear | Clear | - |

| 3 | Urinary nitrite | Negative | Negative | - |

| 4 | Urinary glucose | 3+(28.0) ↑ | Negative (mmol/L) |

| 5 | Urinary protein | 1+(0.30) ↑ | Negative (g/L) |

| 6 | Urine specific gravity | 1.016 | 1.003 - 1.030 |

| 7 | Urinary bilirubin | Negative | Negative (μmol/L) |

| 8 | Urinary ketone bodies | Negative | Negative (mmol/L) |

| 9 | Urine pH | 5.5 | 5.0 - 8.0 |

| 10 | Urobilinogen | Weakly positive | Weakly positive (μmol/L) |

| 11 | Urinary occult blood | 2+(80) ↑ | Negative (Ery/μL) |

| 12 | Urinary leukocyte esterase | ±↑ | Negative (LEU/

| 13 | Urinary Leukocytes | 29↑| <20 (/μL) |

| 14 | Urinary Red Blood Cells | 391↑| <13 (/μL) |

| 15 | Urinary Squamous Epithelial Cells | 3| <47 (/μL) |

| 16 | Urinary Mucus Threads | 6| <137 (/μL) |

| 17 | Urinary Calcium Oxalate Crystals | 1| <14 (/μL) |

| 18 | Uric Acid Crystals | <1| <14 (/μL) |

| 19 | Urinary Struvite Crystals | <1| <14 (/μL) |

| 20 | Casts | 1↑| <1 (/μL) |

**8.3(Emergency) Liver Function - Related Indicators**

| Serial Number | Item Name | Result | Reference Range | Unit |

| 1 | Total Bilirubin | 32.3↑| <21.0 μmol/L

| 2 | Prothrombin Time Activity | 67.0↓| 90.0 - 137.0%|

| 3 | International Normalized Ratio | 1.28↑| 0.9 - 1.1|

| 4 | Activated Partial Thromboplastin Time | 41.6| 30.0 - 45.0 seconds |

| 5 | Thrombin Time | 16.7| <20.0 seconds |

| 6 | Plasma Fibrinogen | 6.09↑| 2.0 - 4.0g/L|

| 7 | D - Dimer | 6500↑| <500μg/L|

**promopt：**

I am an emergency department physician managing an emergency patient. Based on the provided clinical data (history, physical examination, tests, imaging). Please make a diagnosis according to the ICD-10 disease classification, make the most likely diagnosis, and divide it into the main diagnosis and secondary diagnosis (no explanation required).
