# Supplementary material for: Performance evaluation of large language models in the diagnosis of emergency internal medicine diseases: a retrospective study
Source: Front Public Health. 2026 May 8;14:1780425. doi: 10.3389/fpubh.2026.1780425 (PMC13195009; doi:10.3389/fpubh.2026.1780425)
Supplement: Supplementary file 3 [file Table_3.DOCX]

**Supplementary Table S2**

Comparison of main diagnostic accuracy between five LLMs and EDJP in subgroup diseases

**2.1 Digestive system**

| Models | Accuracy | 95% | ^a^*P* value |
| --- | --- | --- | --- |
| EDJP | 84.6% | (0.71-0.98) | >0.05 |
| DeepSeek-V3 | 86.5% | (0.74-0.99) | >0.05 |
| GPT-4o | 84.6% | (0.71-0.98) | >0.05 |
| Grok3 | 92.3% | (0.83-1.00) | >0.05 |
| Gemini-2.0 | 84.6% | (0.71-0.98) | >0.05 |
| Doubao | 82.7% | (0.70-0.95) | >0.05 |

**2.2 Circulatory system**

| Models | Accuracy | 95% | ^a^*P* value |
| --- | --- | --- | --- |
| EDJP | 76.6% | (0.66-0.87) | >0.05 |
| DeepSeek-V3 | 85.9% | (0.75-0.96) | >0.05 |
| GPT-4o | 85.9% | (0.75-0.97) | >0.05 |
| Grok3 | 87.5% | (0.78-0.97) | >0.05 |
| Gemini-2.0 | 71.2% | (0.56-0.88) | >0.05 |
| Doubao | 76.6% | (0.62-0.91) | >0.05 |

**2.3 Urinary system**

| Models | Accuracy | 95% | ^a^*P* value |
| --- | --- | --- | --- |
| EDJP | 54.6% | (044-0.65) | >0.05 |
| DeepSeek-V3 | 63.6% | (0.48-0.79) | >0.05 |
| GPT-4o | 59.1% | (0.46-0.73) | >0.05 |
| Grok3 | 68.2% | (0.51-0.85) | >0.05 |
| Gemini-2.0 | 63.6% | (0.37-0.90) | >0.05 |
| Doubao | 72.7% | (0.55-0.90) | >0.05 |

**2.4 Others**

| Models | Accuracy | 95% | ^a^*P* value |
| --- | --- | --- | --- |
| EDJP | 98.2% | (095-1.00) | >0.05 |
| DeepSeek-V3 | 100% | (1.00-1.00) | >0.05 |
| GPT-4o | 98.2% | (0.95-1.00) | >0.05 |
| Grok3 | 94.6% | (0.87-1.00) | >0.05 |
| Gemini-2.0 | 89.3% | (0.78-1.00) | >0.05 |
| Doubao | 92.9% | (0.86-1.00) | >0.05 |

CI：Confidence interval

P values for main diagnosis accuracy were calculated using Chi-square test, with Bonferroni-corrected pairwise comparisons, p values for differential quality score were obtained from Kruskal–Wallis H test, followed by Dunn’ s post-hoc tests with Bonferroni adjustment。

^a^ refers to comparisons between five LLMs and EDJP
