# Supplementary material for: Performance evaluation of large language models in the diagnosis of emergency internal medicine diseases: a retrospective study
Source: Front Public Health. 2026 May 8;14:1780425. doi: 10.3389/fpubh.2026.1780425 (PMC13195009; doi:10.3389/fpubh.2026.1780425)
Supplement: Supplementary file 4 [file Table_4.DOCX]

**Supplementary Table S4**

In our early tests, we observed that when large language models (LLMs) were given restrictions on the specific number of diagnoses (e.g., "There shall be no more than 2 principal diagnoses and no more than 5 secondary diagnoses"), they often generated diagnoses in a "quota-filling" manner. Specifically, the LLMs would frequently force their outputs to reach the maximum limits: 2 principal diagnoses and 5 secondary diagnoses. This indicates that the LLMs exhibit a sort of "human-pleasing" behavior, or alternatively, a failure to fully comprehend the instructions-resulting in some diagnoses being completely meaningless. In this paper, ChatGPT-4o is used as the test subject to demonstrate this phenomenon.

Furthermore, regarding the expression "Elevated Mean Platelet Volume (D77.9)"-although this indicator reflects a genuine laboratory finding, and clinically, it is necessary to be alert to the fact that gastrointestinal bleeding may be a potential primary cause of this phenomenon, while the possibility of concurrent other etiologies cannot be ruled out, it is important to note that in clinical practice, "increased platelet volume" itself is rarely used as an independent diagnosis, and even less likely is a clear diagnostic association established solely based on this indicator and the D77.9 code.

This expression precisely reflects the limitations of LLMs in medical-related responses: On the one hand, there is obvious information redundancy. Instead of focusing on the core logic that "indicators are merely manifestations and diagnosis must be based on etiologies", it unnecessarily associates non-diagnostic indicators with code combinations. On the other hand, LLMs are incapable of conducting clinically critical "differential diagnosis by exclusion"—they neither further analyze the differentiation points corresponding to different etiologies (such as gastrointestinal bleeding, myeloproliferative diseases, platelet activation, etc.) nor provide prompts for comprehensive judgment based on platelet count, clinical symptoms, and other test results. Ultimately, they can only provide vague descriptions such as "there may be a certain type of condition".

Essentially, such limitations are consistent with the common problems in content generated by large language models: They tend to produce empty and formulaic statements lacking targeting, and incline to cover multiple possibilities through broad expressions to "cater" to the demand for information comprehensiveness. However, they overlook the key requirement of "accurate differentiation and focus on core etiologies" in medical scenarios, resulting in limited clinical reference value of the responses.

**For example：ChatGPT-4o：**
**promopt 1：**

I am an emergency department physician. Please make a diagnosis according to the ICD-10 disease classification, make the most likely diagnosis, and divide it into the main diagnosis and secondary diagnosis.

**The answer 1：**
**1.Primary diagnosis:** K92.1 Gastrointestinal bleeding（presenting with melena）

**2.Secondary diagnoses:**

2.1D50.0 Iron deficiency anemia, secondary to chronic blood loss（severe anemia）

2.2 R60.0 Lower extremity edema, possibly related to hypoproteinemia or anemia

2.3 E87.8 Electrolyte imbalance (hyperchloremia)

2.4 R63.4 Weakness, fatigue (manifested as inability to walk, listlessness)

**promopt 2：**

I am an emergency department physician. Please make a diagnosis according to the ICD-10 disease classification, make the most likely diagnosis, and divide it into the main diagnosis and secondary diagnosis.There shall be no more than 2 principal diagnoses and no more than 5 secondary diagnoses.

**The answer 2：**

**Primary diagnosis**: 1. Gastrointestinal bleeding (K92.2) 2. Severe anemia (D64.9)

**Secondary diagnosis:**

2.1 Hypoproteinemia (E88.0)

2.2 Hyperbilirubinemia (E80.6)

2.3 Coagulation dysfunction (D68.9)

2.4 Hypobicarbonatemia/metabolic acidosis (E87.2)

2.5 Elevated Mean Platelet Volume (D77.9)
